# Recent advancement of novel marine fungi derived secondary metabolite fibrinolytic compound FGFC in biomedical applications: a review

**DOI:** 10.3389/fcimb.2024.1422648

**Published:** 2024-09-18

**Authors:** Lakshmi Jeevithan, Xiaozhen Diao, Jiudong Hu, Jeevithan Elango, Wenhui Wu, Jose Eduardo Mate Sanchez de Val, Saravanan Rajendran, Thanigaivel Sundaram, Suresh Kumar Rajamani Sekar

**Affiliations:** ^1^ Department of Marine Biopharmacology, College of Food Science and Technology, Shanghai Ocean University, Shanghai, China; ^2^ Department of Biomaterials Engineering, Faculty of Health Sciences, UCAM-Universidad Catolica San-Antonio de Murcia, Murcia, Spain; ^3^ Shanghai Sixth People’s Hospital, affiliated to Shanghai Jiao Tong University School of Medicine, Shanghai, China; ^4^ Center of Molecular Medicine and Diagnostics (COMManD), Department of Biochemistry, Saveetha Dental College and Hospitals, Saveetha Institute of Medical and Technical Sciences, Saveetha University, Chennai, India; ^5^ Instituto de Alta Investigacion, Universidad de Tarapacá, Arica, Chile; ^6^ Department of Biotechnology, Faculty of Science & Humanities, SRM Institute of Science and Technology, Kattankulathur, Tamil Nadu, India; ^7^ College of Natural Sciences, Arba Minch University, SNNPR, Arba Minch, Ethiopia

**Keywords:** fungi fibrinolytic compounds, marine metabolites, biomedical applications, signaling mechanism, antithrombotic activities

## Abstract

For several decades, products derived from marine natural sources (PMN) have been widely identified for several therapeutic applications due to their rich sources of bioactive sub-stances, unique chemical diversity, biocompatibility and excellent biological activity. For the past 15 years, our research team explored several PMNs, especially fungi fibrinolytic compounds (FGFCs). FGFC is an isoindolone alkaloid derived from marine fungi, also known as staplabin analogs or *Stachybotrys microspora* triprenyl phenol (SMTP). For instance, our previous studies explored different types of FGFCs such as FGFC 1, 2, 3 and 4 from the marine fungi *Stachybotrys longispora* FG216 derived metabolites. The derivatives of FGFC are potentially employed in several disease treatments, mainly for stroke, cancer, ischemia, acute kidney injury, inflammation, cerebral infarction, thrombolysis and hemorrhagic activities, etc. Due to the increasing use of FGFCs in pharmaceutical and biomedical applications, it is important to understand the fundamental signaling concept of FGFCs. Hence, for the first time, this review collectively summarizes the background, types, mode of action and biological applications of FGFCs and their current endeavors for future therapies.

## 1 Introduction

Marine sources have been continuously explored for biomedical approaches due to their richness of diverse natural products that have excellent anti-inflammatory (Yang et al., 2014; Guo et al., 2016; Xu et al., 2019), antibacterial, antialgal, antilarval (Dobretsov et al., 2013), antifungal (Choudhary et al., 2017; El-Hossary et al., 2017), enzyme inhibitor in marine bacteria (Chellaram et al., 2014; Karthikeyan et al., 2022), antioxidant (; Shindo and Misawa, 2014; Zhong et al., 2019) and anticancer (Deshmukh et al., 2018; Khalifa et al., 2019) activities. At present, 20 drugs from marine natural resources are authorized for commercial application and more than 30 natural products are in different pipeline phases of clinical trials (Haque et al., 2022).

Among the different marine sources, marine micro-organisms play a major role when comes to isolating bioactive substances. They are the major sources of therapeutic drugs such as antithrombotic and anti-inflammatory drugs. In this aspect, marine fungi have been recognized and used for isolating various biologically active secondary metabolites. Interestingly, the secondary metabolites from marine fungi possess excellent antioxidant, antimicrobial, antiviral, anti-inflammatory and anticancer activities (Tarman, 2020; Gonçalves et al., 2022). Therefore, these metabolites are widely used for agriculture, pharmaceutical, cosmetic and medical applications (Gao et al., 2022). The derivatives of secondary metabolites with various structures offer better opportunities to identify novel drugs (Kim et al., 2013; Tian et al., 2015; Youn et al., 2016). Specifically, there is an increasing interest in isolating active fibrinolytic products from marine microorganisms in order to have good pharmacological properties with minimal side effects (Gowthami and Madhuri, 2021; Haque et al., 2022; Papon et al., 2022). To achieve better potential and safety in thrombolysis, scientists have emerged to work on small-molecule natural products, which are supposed to regulate the fibrinolytic pro-enzymes without altering catalytic action (Hasumi et al., 2010; Halland et al., 2015; Sillen et al., 2021). For instance, Shinohara et al. described that the staplabin isolated from the triprenyl phenol compound of *Stachybotrys microspore* stimulated the attachment of plasminogen to U937 cells and fibrin (Shinohara et al., 1996).

Fibrinolysis (prevention of fibrin clot formation) and coagulation are two major processes to control the patency of blood vessels, and thus any improper homeostasis in this process leads to thrombosis *i.e* the excessive activation of coagulation develops thrombi (hemorrhage) or excessive fibrinolysis *i.e* severe bleeding. Fibrin from fibrinogen regulates hemostasis and this process is initiated by thrombin. The major components of fibrinolysis are fibrinogen, plasminogen, activators of plasminogen (urokinase and the tissue type plasminogen activator) and inhibitors of plasminogen (protein C, type 1 and type 2 inhibitors of plasminogen activator). In most cases, the thrombolytic agents for instance alteplase, urokinase, tenecteplase and streptokinase activate plasmin from plasminogen through specific peptide bond cleavage between Val562 and Arg561 for fibrin degradation (Guo et al., 2018; Gao et al., 2021; Gonçalves et al., 2022).

Plasminogen is a precursor protein of plasmin, having a molecular weight of 92 kDa with 791 amino acids and circulates in human plasma about 2 µM (Aisina and Mukhametova, 2014; Joison et al., 2019). During bleeding, the fibrinogen is converted into fibrin in order to stop blood loss by making a 3D mesh network, after that the fibrin clot is degraded by another protein called plasmin, which is formed from plasminogen. During fibrin formation, the major enzymes called tissue-type plasminogen activator (t-PA) and urokinase-type plasminogen activator (uPA) cleave amino acids at Arg561 and Val562 domains in order to activate plasmin (Lijnen, 2001; Schuster et al., 2007). The native circulating Glu-plasminogen (Glu-Plg) exists in two conformations (open and closed) and has seven distinct domains such as a serine protease domain (SP), a Pan-apple domain (Pap), and five kringle (KR1–KR5) domains (containing about 80 amino acid residues) (Xue et al., 2012; Law et al., 2013; Wu et al., 2019). When the fibrin and cell surface receptors interact with kringle domains, it initiates open conformation changes through activation by plasminogen activators (Rijken and Lijnen, 2009; Miles et al., 2021). Previously, it was reported that the interaction of plasminogen with cell surface receptors or fibrin is mainly required for the kringle domains lysine-binding sites (LBSs) (Law et al., 2012; Law et al., 2013). The abnormal plasminogen conformation is possible through the competitive interaction of anti-fibrinolytic agents such as tranexamic acid (TXA) and 6-Aminohexanoic acid (EACA) of lysine analogs with fibrin or cell surface receptors for the interaction to plasminogen LBSs, which further inhibit the activation of plasminogen (McCormack, 2012; Aĭsina and Mukhametova, 2014; Gao et al., 2021).

**Figure 1:**
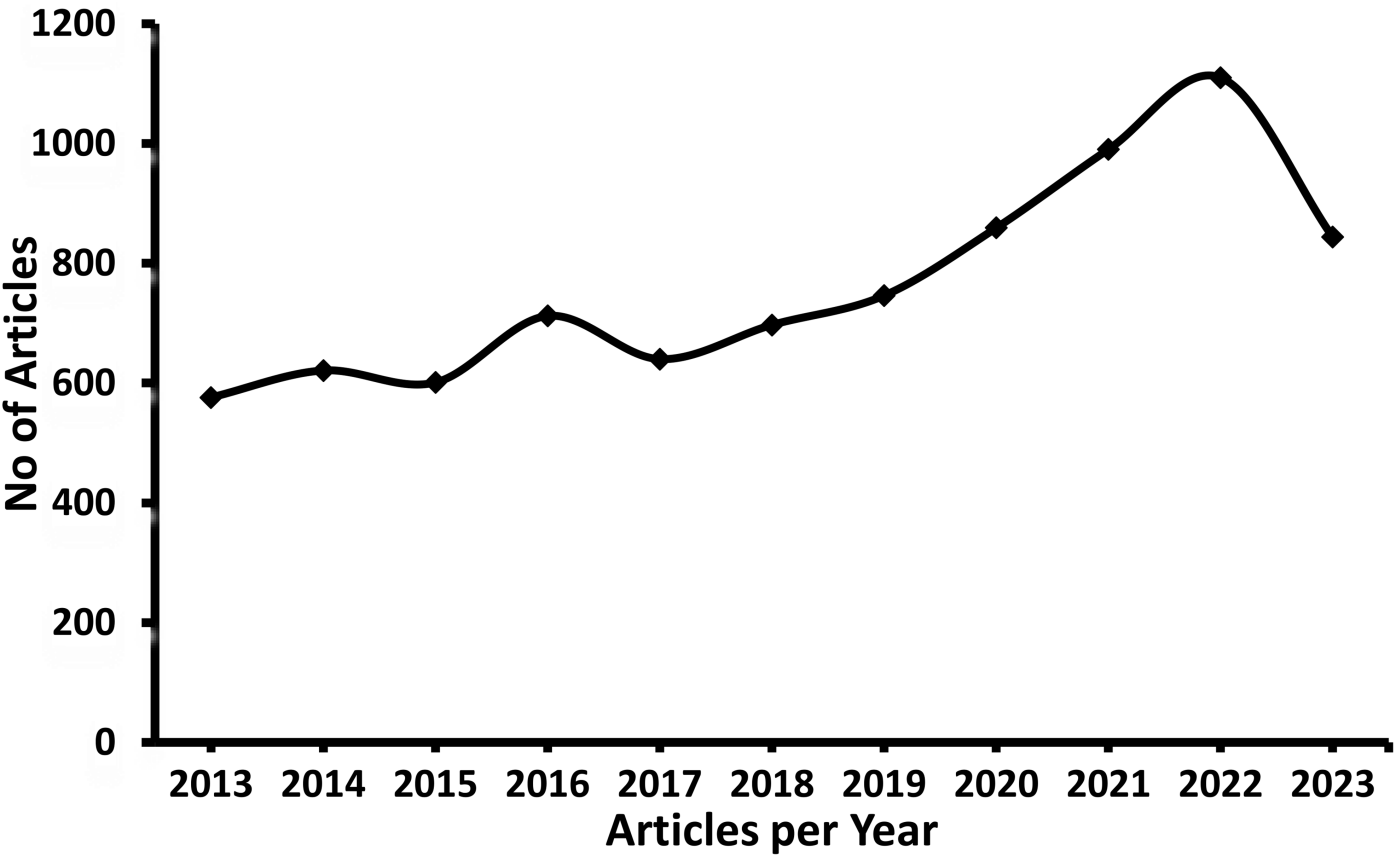
Number of Articles published in Fungi fibrinolytic compounds from marine natural sources (Scopus database).

Our recent studies also reported the isolation and characterization of marine microorganism *Stachybotrys longispora* (*S.longispora*) FG216 derived Fungi fibrinolytic compound (FGFC) with a 869 Da molecular weight (Wang G. et al., 2015; Haque et al., 2022; Yan et al., 2022). FGFC1 is also extracted from *Stachybotrys microspora* (Hasumi et al., 2010; Hasumi and Suzuki, 2021; Shibata et al., 2021). FGFC1 acts as a thrombolytic agent, which reduces the hemorrhagic risk in both *in vivo* and *in vitro*. The tissue distribution studies demonstrated that FGFC1 may be easily absorbed in all tissues in Wistar rats except the brain. FGFC1 activates plasminogen by altering the secondary and tertiary structure and thereby initiates fibrinolytic activity (Wang G. et al., 2015; Gao et al., 2021). Different types of FGFCs were isolated such as FGFC1, FGFC2, FGFC3 and FGFC4 with a wide range of derivatives. In general, FGFCs have been proposed to use in treating various disorders such as cerebral infarction, cancer treatment, thrombolysis and hemorrhage and thus *in vitro* and *in vivo* studies on FGFCs have increased considerably in recent times (**Figure 1**). Consequently, the research on the antithrombotic activity of FGFCs delivered a breakthrough in the treatment of thrombus. Due to their exceptional features, the FGFC is proposed to have the potential to treat different diseases like, pulmonary embolism, ischemic or embolic stroke, drug-induced nephrotoxicity, myocardial infarction, early-stage IgA nephropathy, acute kidney injury (AKI), stroke and renal ischemia/reperfusion (Kemmochi et al., 2012; Huang et al., 2018a; Poirier, 2018; Shibata et al., 2019; Hasumi and Suzuki, 2021; Shibata et al., 2021; Hashimoto et al., 2022; Bailly, 2023). For instance, Guo et al. reported that the catalytic activity of fibrinolysis was improved by FGFC1 via reciprocal activation of pro-uPA and plasminogen (Guo et al., 2018). In addition, Yan et al. investigated the anti-cancer effect of FGFC1 using erlotinib-resistant non-small cell lung cancer and concluded that the anticancer effect of FGFC1 was mediated by the EGFR/PI3K/Akt/mTOR pathway (Yan et al., 2022). In another study, Shibata et al. disclosed the anti-inflammatory and antioxidant activities of FGFC1 in a mouse model of acute kidney injury (Shibata et al., 2021). Hence, it is inevitable to understand the relationship of structure towards the molecular mechanisms of action of fibrinolytic agents. Hence, this review collectively provides fundamental information on FGFCs in various biological functions with their mode of function.

## 2 Types of fungi fibrinolytic compounds

**Figure 2:**
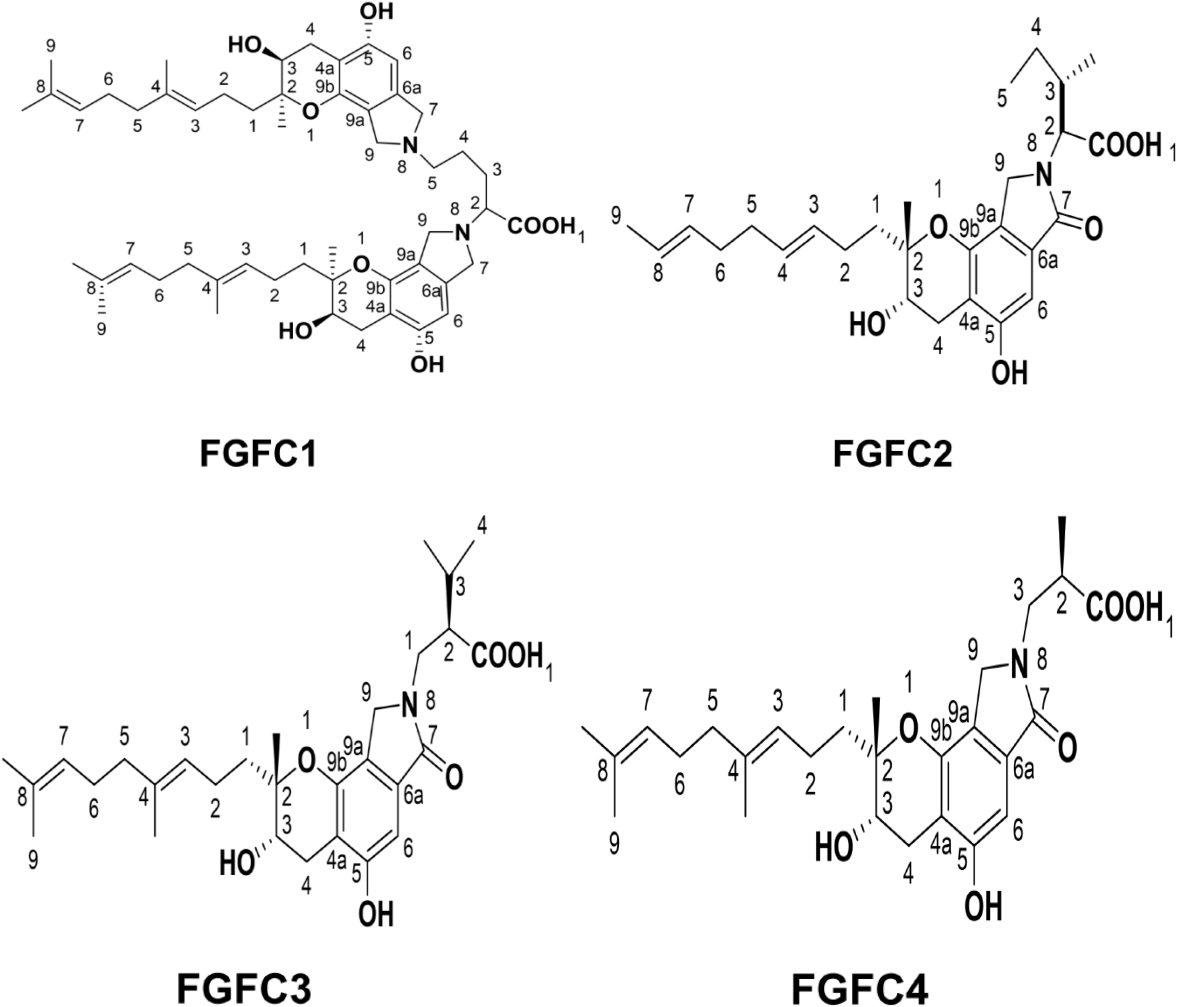
Major types of FGFCs and their structural features.

Up to now, there are different types of FGFCs reported such as FGFC1, FGFC2, FGFC3 and FGFC4 based on their structural variations and functional properties (**Figure 2**). In 1996, FGFC was isolated from fungi *Stachybotrys microspora* IFO 30018 by Kohyama et al. and found that FGFC accelerated plasminogen-fibrin binding action by 20-30% compared to the basic core of the SMTP family, a triprenyl phenol and staplabin (Shinohara et al., 1996; Kohyama et al., 1997; Hu et al., 2000; El Maddah et al., 2024). Bridging of ornithine with two staplabin cores of FGFC would increase activation of urokinase-catalyzed plasminogen, fibrin binding of plasminogen, and plasminogen/urokinase-mediated fibrinolysis (Hu et al., 2000; Hang et al., 2022). Our previous studies also explored the isolation and characterization of marine fungi *S. longispora* FG216 metabolite derived FGFCs (FGFC1, FGFC2, FGFC3 and FGFC4) (Wang G. et al., 2015; Guo et al., 2016; Yan et al., 2022).

FGFC1 is the most studied isoindolone derivative of Fungi fibrinolytic compound and can be extracted from different marine microorganisms (Wang G. et al., 2015). For instance, FGFC1 with more than 98% purity was extracted from the methanol extracts of *S. longispora* FG216 (Yan et al., 2022) and reported to increase plasmin activity from 2.05 to 11.44 folds with 0.1–0.4 M FGFC1, respectively through activation of Lys-plasminogen (Lys-Plg) and Glu-Plg (Wang G. et al., 2015). Guo et al. reported that the FGFC1 altered the stimulation of reciprocal activation system-dependent enzymatic kinetic parameters by a single-chain urokinase-type plasminogen activator (scu-PA) (Guo et al., 2018). The carbon skeleton of FGFC1 was biosynthesized by Wang et al. with a yield rate of 9.92 and 9.6 g/L along with FGFC2 and FGFC3 *via* the fermentation process contributing to precursor regulation and inhibitor pathways such as mevalonate and shikimate pathways. Yan et al. proved the thrombolytic activity of FGFC1 for thrombus treatment without significant risk of hemorrhagic activity by conducting the experiments on pharmacodynamic action of FGFC1 in both the hemolytic and the thrombolysis by fibrinogenolysis (Yan et al., 2015). In another study, Gao et al. fabricated a chromogenic substrate-based novel fibrinolytic system comprising plasminogen and a scu-PA to evaluate the fibrinolytic activity of FGFC1 (Gao et al., 2022). Also, the tissue distribution and pharmacokinetics of FGFC1 were examined by Su et al. by intravenous administrations of fluorescence-labeled FGFC1 in the Wister rat model. They found that FGFC1 was instantly circulated in all tissues except the brain, having higher absorption in the liver and evidencing enterohepatic circulation of FGFC1 (Su et al., 2013).

Gao et al. explored the mode of interaction and binding sites of plasminogen with FGFC1 by using molecular docking and *in vitro* experiments (Gao et al., 2021). Empirical evidence proved the exceptional potential of the marine fungus *S. longispora* FG216 isolated FGFC1 in fibrinolytic, thrombolysis and hemorrhagic activity. FGFC1 has a unique two symmetrical carbon chains chemical structure linked with ornithine, and the carbon skeleton of FGFC1 could be biosynthesized by the precursor regulation and pathway inhibitor process involving the mevalonate and shikimate pathways. The integration of key precursor ornithine into the carbon skeleton of FGFC1 could produce two essential intercedes of FGFC1 such as FGFC2 and FGFC3. In FGFC1, the two symmetrical carbon chains are linked through an ornithine molecule and biosynthesis of FGFC1 involves combining one *L*-ornithine into two FGFC3, which has intermediate compound FGFC2 during the first step of the process i.e insertion of a molecule of L-ornithine with fist FGFC3 molecule. Therefore, the biosynthesis of FGFC1 involves three major compounds such as FGFC2, FGFC3, and *L*-ornithine (Wang M. et al., 2015).

FGFC2 is one of the critical intermediates that arise from the integration of key precursor ornithine into the carbon skeleton of FGFC1. As discussed earlier, the major differences between different FGFC is related to the changes in their functional groups and side chains. As a concept of proof, the major differences between FGFC1, FGFC2 and FGFC3 were studied in terms of their chemical structure by Wang et al. (Wang M. et al., 2015). Previously, FGFC2 (isoindolone, Fungi fibrinolytic compound 2) was isolated by Guo et al. from an organic extract of *S. longispora* FG216 strain using ethyl acetate fraction along with LL-Z1272β and ergosterol. This study investigated *in vitro* fibrinolytic activities of FGFC2 and ergosterol and reported EC_50_ values about 108.16 and 156.30 μmol/L for FGFC2 and ergosterol, respectively (Guo et al., 2016).

As discussed earlier, FGFC3 is an intermediate compound when key precursor ornithine is inserted into the carbon skeleton of FGFC1. Previously, the structural difference and their relationships among four FGFCs were inferred after the fermentation bioprocess and concluded that one molecule of FGFC1 was synthesized by inserting a molecule of L-ornithine into 2 molecules of FGFC3, on the other hand, a molecule of FGFC2 was synthesized by inserting a molecule of L-ornithine into a molecule of FGFC3. Therefore, FGFC3 plays a major role in synthesizing FGFC1 and FGFC2 (Wang M. et al., 2015).

Previously, Yin et al. biosynthesized four types of FGFCs such as FGFC4, FGFC5, FGFC6 and FGFC7 by genome mining of isoindolinone biosynthetic gene cluster in Stachybotrys (Yin et al., 2017). In addition, FGFC1-like compounds such as bromo-benzyl derivative (FGFC6) were synthesized to obtain a novel antithrombotic non-cytotoxic agent (Yin et al., 2017). Previously, the FGFC7 model compound was isolated from *S. longispora* FG216, which originates biosynthetically by inserting L-threonine derived isoindolinone core and orsellinic acid. In addition, the biosynthesized FGFC7 could perform the fibrinolytic activity by activating prourokinase (pro-uPA) to produce active form plasmin from zymogen protease plasminogen for the disintegration of blood clot (Yin et al., 2017).

## 3 FGFCs derivatives

Apart from different types of FGFCs, several structural derivatives were also synthesized by including aliphatic (ethyl, methyl, and *n*-propyl) groups to C-2, C-2′ and C-1″ positions in FGFC1 for F1–F3 derivatives, respectively. For F4, F5, F6 and F7 derivatives, benzyl derivatives into the 4-position of benzyl scaffold, trifluoromethoxy group into the para position of benzene ring, 4- position of Br into para and 3 position of Br into ortho position of FGFCs were introduced, respectively (Wang et al., 2021a). These groups were introduced to FGFC1’s C-1″, C-2, and C-2′ positions. Studies showed that the fibrinolytic activity of derivatives F1-F3 and their EC_50_ values might be related to their aliphatic chain length and surprisingly, F1–F3 aliphatic groups showed 1.3–2.0 folds more fibrinolytic activity than FGFC1, suggesting alkyl moieties substitution in C-1″, C-2, and C-2′ increased fibrinolytic activity (Wang et al., 2021a). Interestingly, the FGFC1’s fibrinolytic activity was accelerated via Methyl substituted derivatives F1 (EC_50_ value of 59.7 μM), benzyl derivatives F4–F7, F4 (EC_50_ - 82.8 μM) and F6 (EC_50_ - 42.3 μM), and 4-Br-containing F6 (EC_50_-42.3 μM). On contrary, the fibrinolytic activity was decreased by inserting the trifluoromethoxy group into the para-position of the benzene ring of derivative F5, however, the fibrinolytic activity was retained by interchanging Br atom to *ortho* position from para to get 3-Br-containing F7(EC_50_ = 119.6 μM for F7 *Vs* EC_50_ = 115.0 μM for FGFC1). In addition, shifting the ortho position of bromo groups from para remarkably changes urokinase-activating efficiencies with EC_50_ of F7 (119.6 μM) and F6 (42.3 μM). All these findings suggested that shifting or inserting the functional groups in FGFC1 could potentially improve the fibrinolytic activity, which was admirable in in-depth research and exploration (Wang et al., 2021a).

## 4 Mode of action

**Figure 3:**
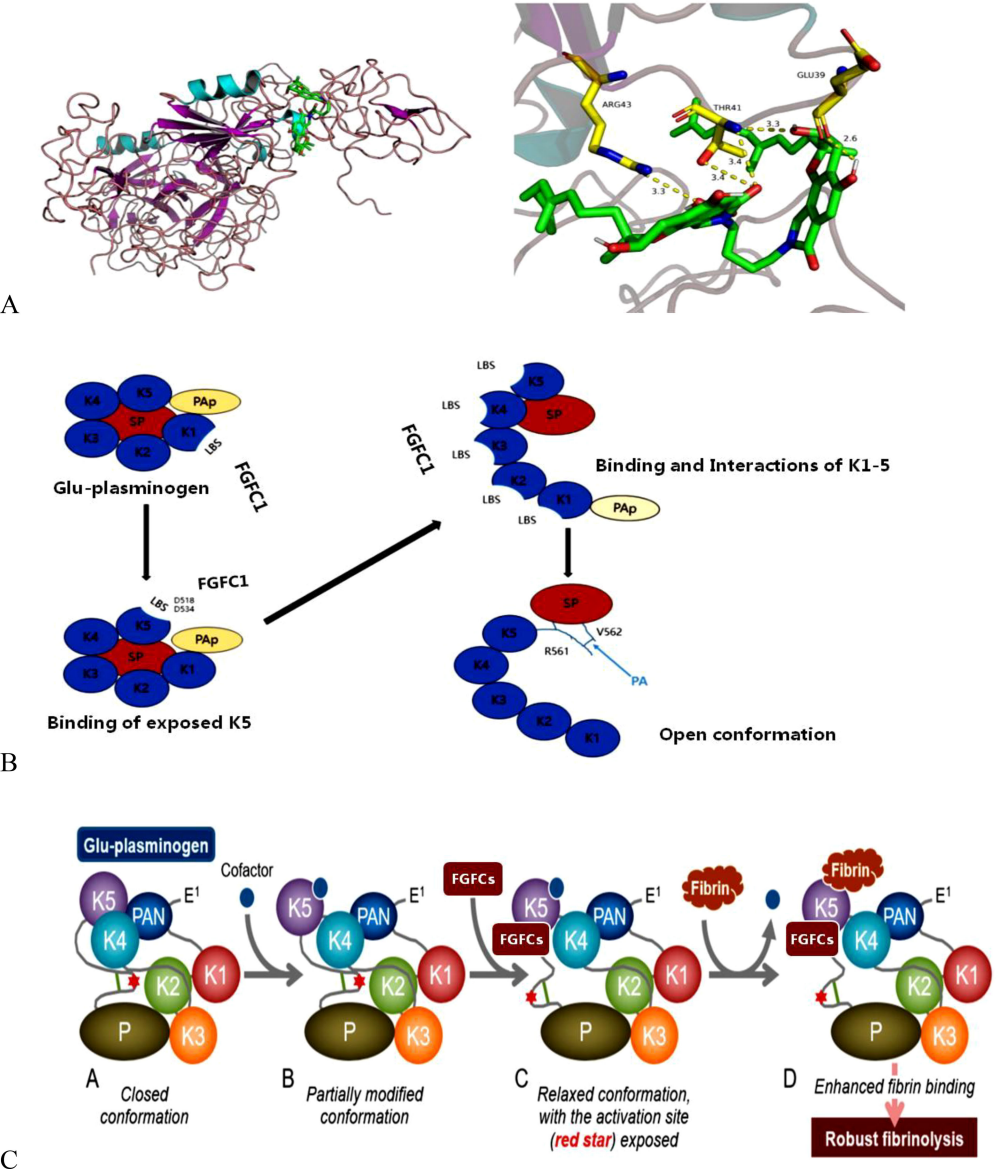
Schematic illustration of conformational interaction between plasminogen and FGFCs **(A)**, the activation mechanism of plasminogen by FGFCs **(B)**, and Open-closed conformation changes of plasminogen during FGFCs binding **(C)** (Gao et al., 2021; Hasumi and Suzuki, 2021).

FGFC is a well-known thrombolytic compound and apparently, it interacts with plasminogen to initiate thrombolysis. The action of FGFC starts with their primary interaction with plasminogen, where the binding sites play a crucial role in signaling pathways. It is reported that lysine-binding sites (LBSs) are the major points for the interaction of FGFC and plasminogen, which was evidenced by the molecular docking experiment through inhibiting FGFC1’s fibrinolytic activity by TXA and EACA (Matsumoto et al., 2015; Gao et al., 2021). The binding of FGFC with plasminogen at LBSs activates plasminogen to expose the open conformation. Further, the molecular docking studies revealed that ligands (EACA, FGFC1) docking with receptors (KR1–KR5) was achieved via hydrophobic and hydrophilic interactions. Both Lys-Plg (open) and Glu-Plg (closed) have the opposite conformation and the open position of Lys-Plg is achieved by cleavage between Lys77 and Lys78 in intact plasminogen that resulted in the PAp domain exclusion from Lys-Plg. Interestingly, the fibrinolytic activity of FGFCs mediated by Lys-Plg is more efficient even at a lower concentration of FGFC (0.072 mm) than FGFC1’s fibrinolytic activity mediated by Glu-Plg (**Figure 3**).

**Figure 4:**
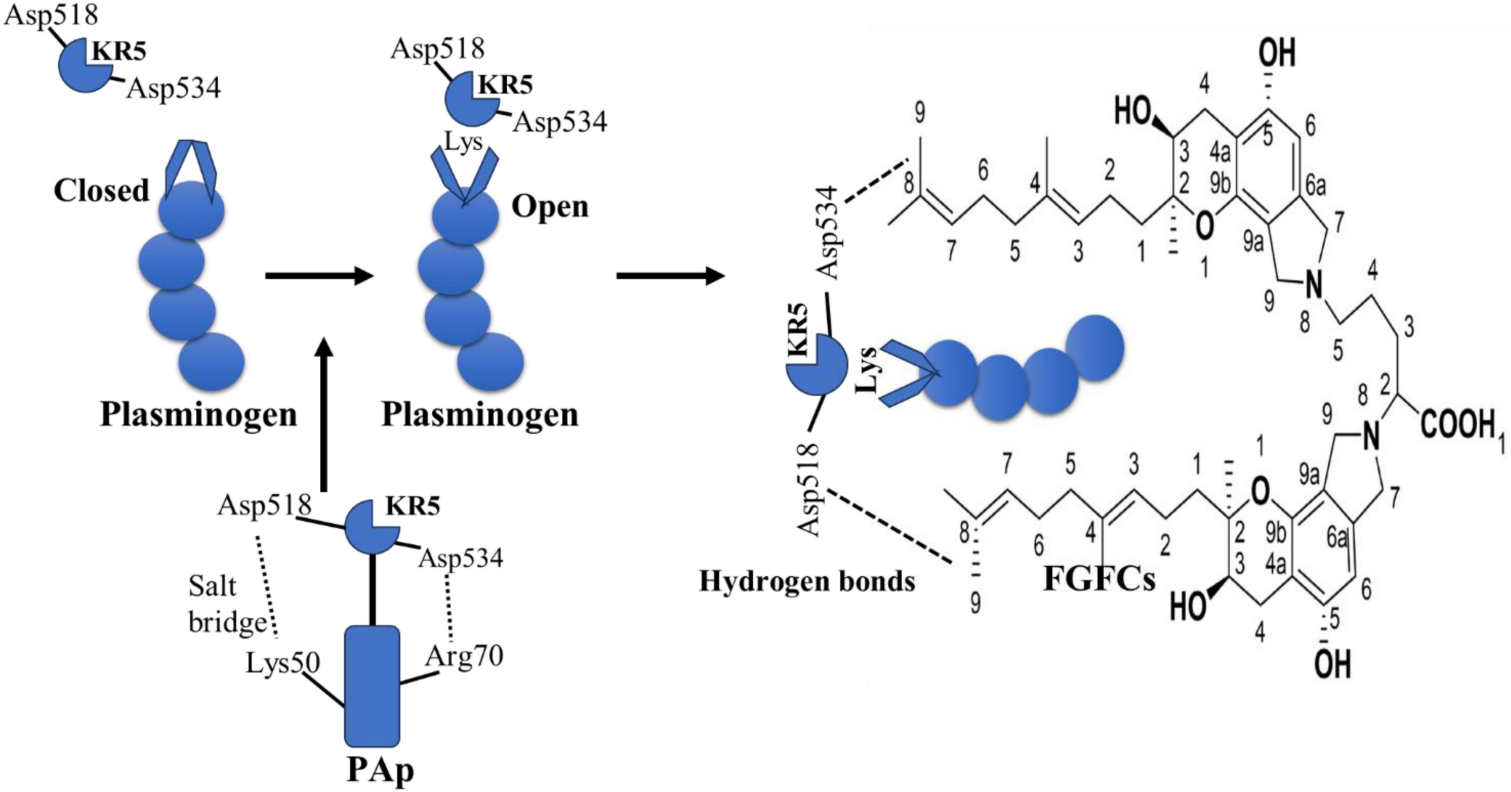
Molecular interaction of KR5 shedding from Pap and its confirmational changes in FGFCs (Gao et al., 2021).

The KR5 shedding away from the PAp is exposed to a closed plasminogen and binds with an external lysine, thereby initiating structural changes. In the above concept, the key interaction is the formation of a salt bridge between Asp518 of KR5 and Lys50 of the PAp domain and coordination between Arg70 of the PAp domain and Asp534 of KR5 (**Figure 4**). Our previous molecular docking results concluded that the hydrogen bond is formed between the group of FGFC1 and Asp518 and Asp534 of KR5 during FGFC1 and KR5 interaction, whereas hydrogen bonds were formed with Thr41, Glu39, and Arg43 residues during FGFC1 and plasminogen interaction (Gao et al., 2021). From the above findings, the overall mode of action of FGFC is summarized in two ways:

FGFC1 behaves as fibrin or cell surface at low concentration and initiates the following mechanism: 1) At first, FGFC1 primarily interacts with LBS of KR1, substantially presents the KR5 shedding from PAp to closed plasminogen. 2) Consequently, FGFC1 triggers a structural rearrangement and conformational change in plasminogen by interacting with Asp534 and Asp518 of the exposed KR5. 3) This leads to the exposition of the more kringle domains LBSs, igniting migration of Pap domain, which creates a chance of more binding between FGFC1 and LBSs. 4) These kringle domains sequence interactions conduct the open conformation in order to functionalized by plasminogen activators. On contrary, FGFC1 acts as EACA or TXA at high concentration due to the presence of the same functional groups (Nε side chain and carboxyl group) and plasminogen binding sites. Also, the surplus FGFC1 suppressed the activation of plasminogen due to the formation of abnormal conformation from intemperance binding of specific sites of plasminogen (Gao et al., 2021).

## 5 Biological application

### 5.1 In thrombolysis and hemorrhagic activities

FGFC is well-known compounds for thrombolysis or fibrinolysis (breakdown of the fibrin in blood clots) by activating plasminogen. Our previous study investigated the low-weight fibrinolytic pyranoindole molecule on Fluorescein isothiocyanate (FITC)-fibrin *in-vitro* degradation study and acute pulmonary thromboembolism *in vivo* study. The study results concluded that FGFC1 degraded FITC-fibrin based on the different concentrations (5-25 μ M) and dissolution of extrinsic FITC-fibrin induced blood clots with 5 mg/kg or above (Yan et al., 2015). Our other study investigated the effect of FGFC1 on clot lysis, structure, and activation of plasminogen and evidenced fibrinolytic activity of FGFC1 by increasing the protofibrils number within fibrin fiber via releasing fibrinopeptide B, D-dimer concentration in lysate, and plasma *p*-nitroaniline’s generation rate to form a thinner region and laxer networks of clots divided by big pores (Gao et al., 2023).

Guo et al. studied the enzymatic kinetic properties of FGFC1 on plasminogen and scu-PA (Guo et al., 2018). Another study reported the molecular docking and fibrinolytic activity of FGFC1 using scu-PA (Gao et al., 2021). FGFC1 isolated from *S. longispora* FG216 accelerated generation of plasmin activity at 0.1–0.4 M determined from Glu-Plg and *Lys-Plg* activation *in vitro* and concluded that FGFC1’s fibrinolytic activity was achieved through plasminogen and scu-PA activation (Wang G. et al., 2015). Shinohara et al. reported that staplabin treatment increased the interaction of plasminogen with fibrin in U937 cells (Shinohara et al., 1996).

The effect of FGFC1 in the activation of plasminogen depends on their cofactors like gangliosides, oleic acid and phospholipids. It is suggested that the long-chain alkyl and alkenyl group of these cofactors would promote thrombolysis of FGFC1 and the cofactor molecule binding to the oleic acid binding site of plasminogen’s k5 domain is the major mechanism in thrombolysis (Koyanagi et al., 2014). In addition, different *N*-linked side chains of FGFCs exhibited varying activity in plasminogen activation. For instance, structurally resembled FGFC compounds with carboxy- and/or hydroxy-substituted aromatic are more active, whereas highly hydrophobic or hydrophilic functions are basically inactive (Hasumi and Suzuki, 2021). Accordingly, the thrombolytic ability of FGFCs plays a major role in other pathophysiological conditions since the plasminogen/plasmin system is a fundamental process for pericellular proteolysis, which is widely involved in several biological processes such as embryogenesis, angiogenesis, tissue remodeling, tumor growth/metastasis and wound healing (Castellino and Ploplis, 2005; Danø et al., 2005; Lund et al., 2006; Hasumi and Suzuki, 2021). As a concept of proof, the FGFCs were widely used in hepatitis, Crohn’s disease, nephritis, Guillain–Barré syndrome, metabolic disease, ulcerative colitis, cancer cachexia, tumor angiogenesis and tumor growth treatment (Weimin, 2003; Matsumoto et al., 2014).

### 5.2 Neuroprotective effect

**Figure 5:**
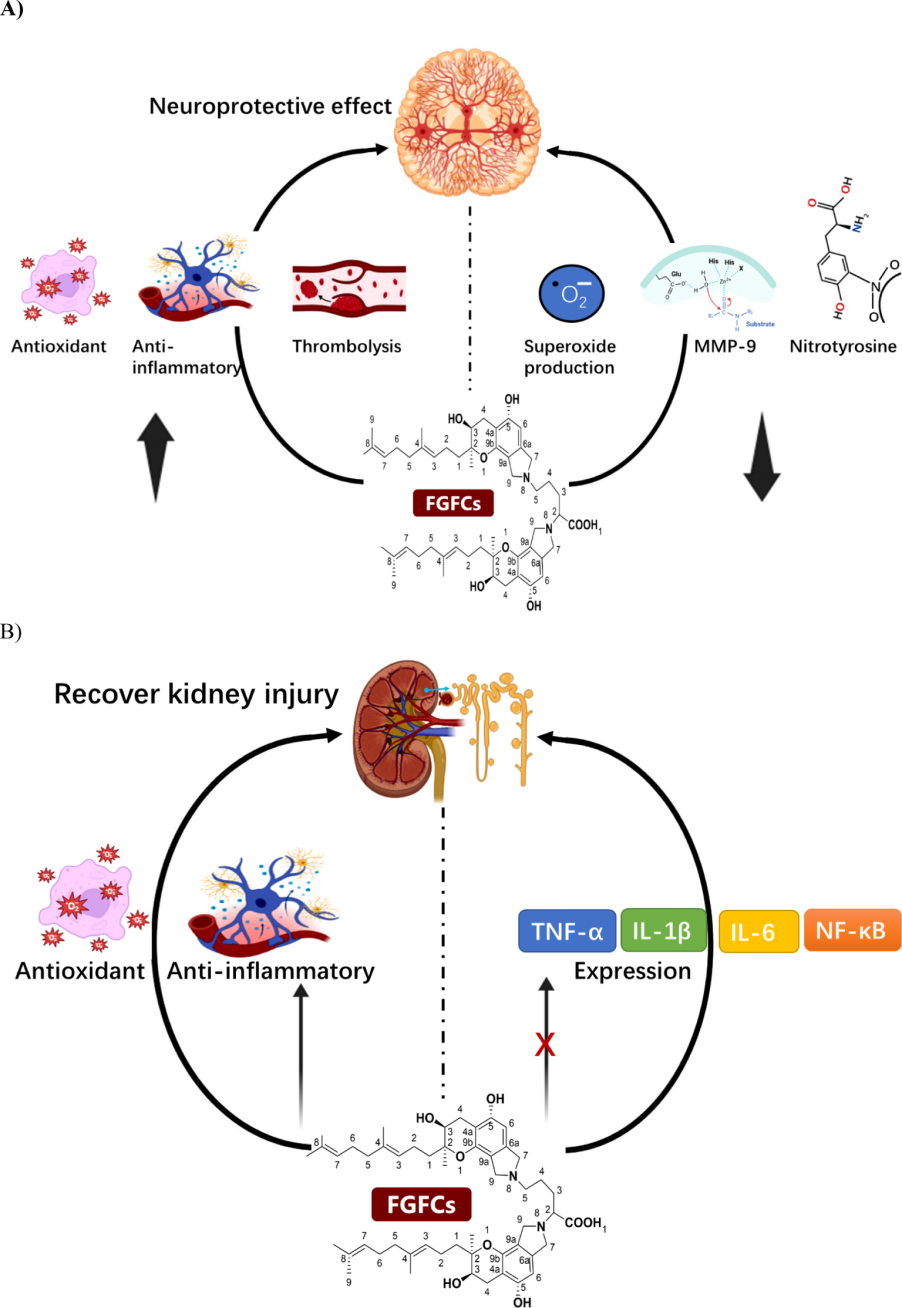
**(A)** The mode of the mechanism of Neuroprotective effect of FGFCs, **(B)** The protective mechanism of FGFCs in Kidney injuries.

FGFCs showed promising effects in reducing size of infarction, clot, and edema in the embolic stroke cynomolgus monkeys model (Poirier, 2018) (Bailly, 2023). Several studies reported the effect of FGFCs on cerebral infarction using several types of ischemic models in rodents and monkeys (Hashimoto et al., 2010; Akamatsu et al., 2011; Shibata et al., 2011; Hashimoto et al., 2014; Sawada et al., 2014; Huang et al., 2018a; Suzuki et al., 2019). The pathophysiological and therapeutic effects of FGFC1 were reported to be higher than *t-PA* by Hashimoto et al (Hashimoto et al., 2010). The antioxidant activity of FGFC1 could enhance the functions in the treatment of embolic infarction (Shibata et al., 2018). Therefore, the effect on cerebral infarction is based on the triad of anti-oxidant activities, anti-inflammatory, and thrombolytic activities (**Figure 5**) (Akamatsu et al., 2011; Hashimoto et al., 2014; Matsumoto et al., 2014; Shibata et al., 2021).

The intrinsic neuroprotective effect of FGFC1 using ischemia/reperfusion injured brain of a rat model was investigated by Akamatsu et al. and disclosed that FGFC1 suppressed the expression of MMP-9, nitrotyrosine and superoxide production in transient focal cerebral ischemia and thereby attenuated ischemic neuronal damage i.e. intrinsic-neuroprotective effect on ischemia injury (Akamatsu et al., 2011). In another study, the embolic stroke mouse model with acetic acid-induced embolus brain after thrombolysis with FGFC1 and t-PA concluded that FGFC1 could minimize the occurrence of embolic stroke by inhibiting proinflammatory gene overexpression and promoting anti-oxidative effect (Hashimoto et al., 2014). Similarly, Hashimoto et al. investigated the protective mechanism of action of FGFC1 along with t-PA in the gerbils cerebral infarction embolic model. They reported that FGFC1 (10 mg/kg) improved the infarction area more efficiently than t-PA. Both groups enhanced antioxidant and anti-inflammatory effects and hence increased the time-dependent neurological score (Hashimoto et al., 2010). Another finding reported that the neuroprotective effect of FGFC1 on ischemia/reperfusion was exerted by suppressing superoxide radicals production and MMP-9 expression, mainly involving hemorrhagic transformation and secondary brain injury (Akamatsu et al., 2011; Ito et al., 2014). Administration of 10 mg/kg FGFC1 for 30 min in a thrombotic middle cerebral artery occlusion monkey’s model induced by photochemical could significantly decrease hemorrhagic transformation by 51% by alleviating the neurologic deficit with minimal infarct size (Sawada et al., 2014). Similarly, the neuroprotective effect of FGFC1 and t-PA toward the neurovascular unit was demonstrated in an intravenous administered mice model with transient middle-cerebral artery-occlusion by decreasing the hemorrhage volume (Huang et al., 2018b).

After achieving a great success rate and convincing findings in higher primates, the research on FGFCs has been taken to the next level in human phase I trials. (Moritoyo et al., 2023) for the first time investigated the anti-inflammatory profibrinolytic effect of FGFCs family triprenyl phenol in human healthy volunteers through pharmacokinetics and pharmacodynamics. Their findings summarized the below major findings: administration of FGFCs at a single dose of different concentrations ranging from 0.3 to 360 mg did not produce major adverse effects, no sign of bleeding determined by biochemical testing and brain imaging analysis, did not reduce α2-antiplasmin and fibrinogen levels and promising pharmacokinetic benefits in human (Hasumi and Suzuki, 2021). In continuation, the phase II human clinical trial was performed in ischemic stroke patients (JapicCTI-183 842) and evidenced the major breakthrough in that a single dose injection of FGFCs achieved 0% of symptomatic intracerebral hemorrhage, whereas 3% was observed for placebo. From the above clinical evidence, in 2021 Biogen, a US-based pharma company started to manufacture FGFCs (also named TMS-007 or BllB-131), an excellent thrombolytic drug for acute ischemic stroke persons. The therapeutic effect of FGFC1 in ischemic stroke is mainly achieved by decreasing the 8-OHdG and 4-NHE expression (Hasumi and Suzuki, 2021). At present, initiatives with large multicenter, dose-ranging, interventional, and randomized trials have been started by Biogen to evaluate the effects of BIIB131 on arterial revascularization, angiographic reperfusion and infarct evolution on acute and 90-day patients (including 760 patients) with ischemic stroke (NCT05764122) (Hasumi and Suzuki, 2021).

### 5.3 In acute kidney injury

AKI is a pathological condition where rapid reduction in renal function due to retaining wastes and toxins in the kidney that potentially affect electrolyte and acid-base balance (Bonventre and Yang, 2011). Su et al. investigated pharmacokinetics and the tissue distribution of FGFC1 administered in Wistar rats through intravenous bolus administration and concluded that elimination half-lives (*t*_1/2_) of FGFC1 were 21.51 ± 2.17 and 23.22 ± 2.11 min, systemic clearance was 0.023 ± 0.002, 0.022 ± 0.002 ((mg/kg)/(μg/mL)/min) for 10 and 20 mg/kg, respectively (Su et al., 2013). The study conducted by Kemmochi et al. investigated the potential benefits of SMTP-7 in treating early-stage IgA nephropathy caused by nivalenol in female BALB/c mice. The researchers found that administering SMTP-7 at a dosage of 10 mg/kg could potentially slow down the progression of IgAN induced by nivalenol by preventing the local accumulation of IgA in the glomerular mesangium (Kemmochi et al., 2012). In the previous study, the treatment of FGFC1 in an AKI mouse model improved the renal functional properties such as blood urea nitrogen, serum creatinine and fractional excretion of sodium, to minimize the renal tubule damage with excellent anti-oxidative and anti-inflammatory activities by inhibiting IL-1β, TNF-α, IL-6, and NF-κB (**Figure 5**) (Shibata et al., 2021). The therapeutic effect of FGFC1 in AKI model mice was investigated by Shibata et al. and reported that FGFC1 recovered renal function by protecting renal tubules against damage via concentration-dependent (0.01, 0.1, 1, 10 mg/kg). The FGFC1 sheltered the AKI model mice by exhibiting anti-inflammatory and antioxidant activities (Shibata et al., 2021).

Among Acute kidney injuries, IgA nephropathy is considered a major chronic glomerulonephritis currently, which leads to a gradual renal function decline in those affected by renal disease (30%) (Mubarak, 2011; Tesfaye et al., 2013; Pop-Busui et al., 2017). The therapeutic effect of FGFC1 on IgA nephropathy was conducted by Kemmochi et al. and found that FGFC1 at 10 mg/kg decreased the IgA deposition in the glomerular mesangium, which may propose to have an inhibitory effect of FGFC1 in IgA nephropathy progression (Kemmochi et al., 2012; Hang et al., 2022).

### 5.4 In cancer

Over the last few decades, natural products have been recognized as a promising and valuable reservoir of potential anti-cancer drug candidates due to their ability to target multiple pathways and address the limitations of single-agent therapy, including side effects and drug resistance. In our previous study, FGFC1 was treated with Erlotinib-Resistant Non-Small Cell Lung Cancer (NSCLC) cells and the study report showed that FGFC1 triggered the NSCLC cells apoptosis based on their concentration, and downregulated the phosphorylation of protein EGFR, protein kinase B (Akt), phosphatidylinositol 3-kinase (PI3K), and mTOR in H1975 cells (Yan et al., 2022). One of the novel approaches of anticancer drugs for cancer therapy is to inhibit the angiogenesis of abnormal cells, which regulates the irregular proliferation and differentiation of cells (Ismail et al., 2021; Hang et al., 2022; Bailly, 2023; Liu et al., 2023). Hence, inhibiting the cell cycle of tumor cells can hinder cell growth and trigger programmed cell death. Programmed cell death, known as cell apoptosis, plays a crucial role in tumor formation and progression. Various mechanisms, including gene regulation and signal transduction, are implicated in this process. Stimulating cell apoptosis proves to be an efficacious approach for treating cancer.

**Figure 6:**
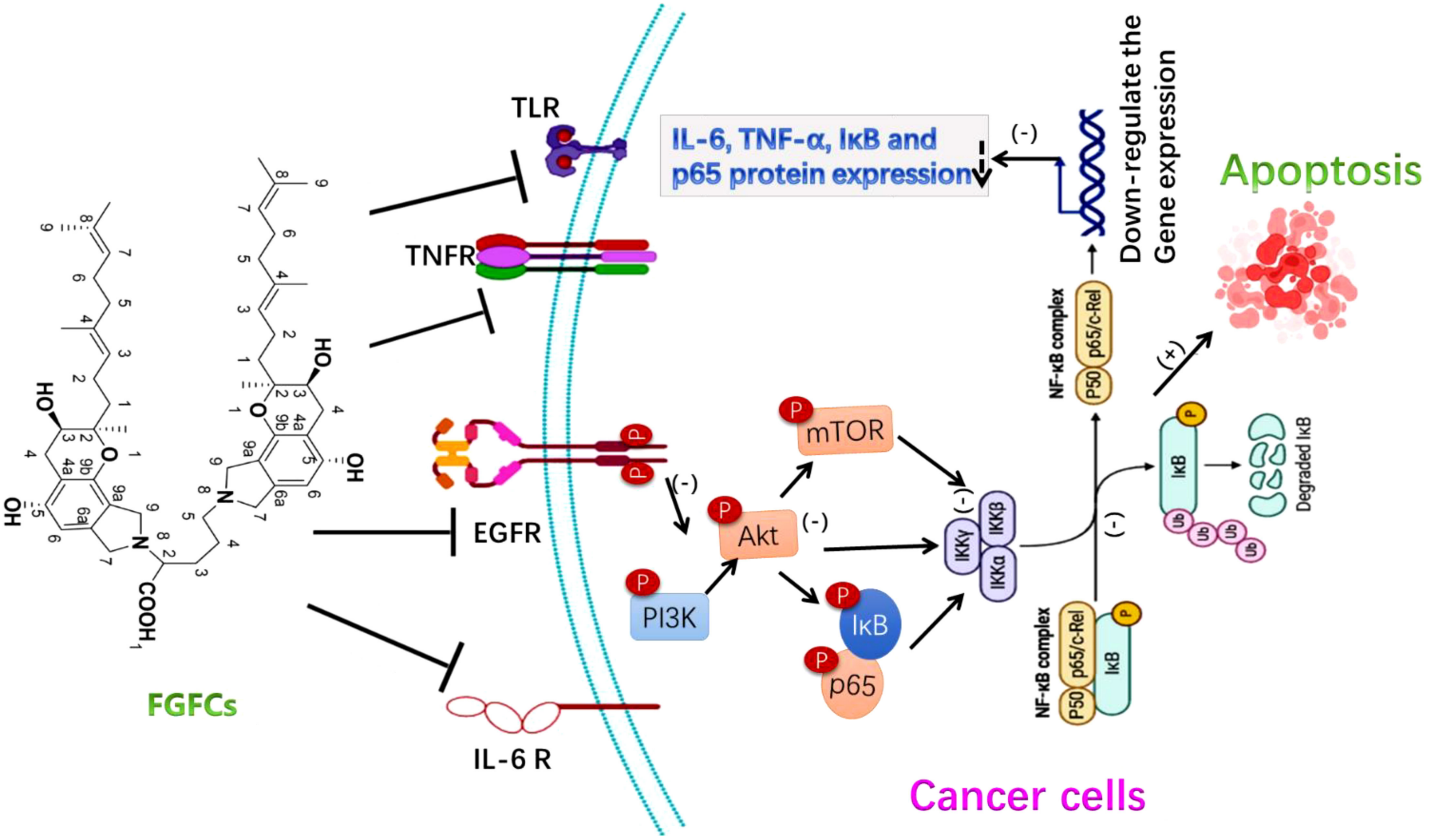
The molecular signaling mechanism of FGFCs in Anticancer activity (Yan et al., 2021; Feng et al., 2022; Yan et al., 2022).

The anticancer ability of FGFC1 was investigated in NSCLS and EGFR-mutant NSCLC cells and reported that FGFC1 inhibited cell growth and cancer cell migration by down-regulating the NF-kB signaling pathway via signaling protein expressions such as IL-6, TNF-α, p-IκB, and p-p65. The FGFC1 treatment decreased the cell count from 1580 to 231, providing further evidence of FGFC1’s ability to inhibit cell growth in EGFR-mutant PC9 cells (Feng et al., 2022). Also, the intraperitoneal treatment of FGFC1 (10 mg/kg) reduced dermatologically placed PC9 cell growth via NF-κB signaling pathway inhibition in tumor xenografted mice and thus reduced tumor volume (Yan et al., 2021; Feng et al., 2022). As a concept of proof, Yan et al. investigated the molecular signaling mechanism of FGFC1 in cancer treatment using a chemoresistant xenograft model and reported the mitochondrial dysfunctions associated with the dose-dependent effect of FGFC1 in restricted proliferation and increased NSCLC H1975 cells apoptosis through directly binding to the silenced EGFR kinase. Also, the treatment of FGFC1 both *in-vivo* and *in-vitro* accelerated the ROS and decreased the EGFR/Akt/PI3K/mTOR pathway (**Figure 6**) (Yan et al., 2022). The adaptable nature of FGFC1 in stability, solubility and oral drug release proves as a good drug candidate for the promising treatment of thrombolytic and cancer.

### 5.5 Inflammatory disease treatment

Inflammation is caused by normal tissue damage, which affects the blood coagulation and regulation system (thrombotic complications). For instance, pulmonary embolism and vein thrombosis were developed three-fold higher in patients suffering from inflammatory bowel disease (Wakefield et al., 2008; Olivera et al., 2021; Gala et al., 2022). A recent study reports using the renal ischemia or reperfusion-induced AKI rodent model showed that the FGFC1 with 10 mg/kg lowered or inhibited pro-inflammatory cytokines (IL-1β and TNF-α, respectively) expression (Shibata et al., 2021; Hashimoto et al., 2022). Thereby, researchers initiated to investigate the effective role of FGFC1 in treating ischemia/reperfusion and inflammatory diseases. In this sense, the researchers have been targeted to inhibit the activity of soluble epoxide hydrolase (sEH), since sEH produces an inflammatory response by activating lipid signaling molecule hydrolyzation (Harris and Hammock, 2013; Morisseau and Hammock, 2013). Interestingly, FGFC1 is proven as an inhibitor of sEH by acting against sEH activation through its geranyl methyl side-chain (Blunt et al., 2016; Hang et al., 2022). More specifically, the geranyl methyl side-chain interacts with the C-terminal domain of sEH, which catalyzes the hydrolysis of an endogenous signaling molecule involved in anti-inflammation, i.e. epoxyeicosatrienoic acids. Therefore, the interaction of the geranylmethyl side-chain and C-terminal domain of sEH inhibited the hydrolysis of epoxyeicosatrienoic acids (Thomson et al., 2012; Wang et al., 2021b) (Ulu et al., 2013; Matsumoto et al., 2014). In most cases, the anti-inflammatory effect of FGFC1, which might account for the inhibition of sEH is closely investigated along with the purpose of other therapeutic treatments such as cancer, cerebral infarction and AKI (Matsumoto et al., 2014; Shibata et al., 2021).

**Figure 7:**
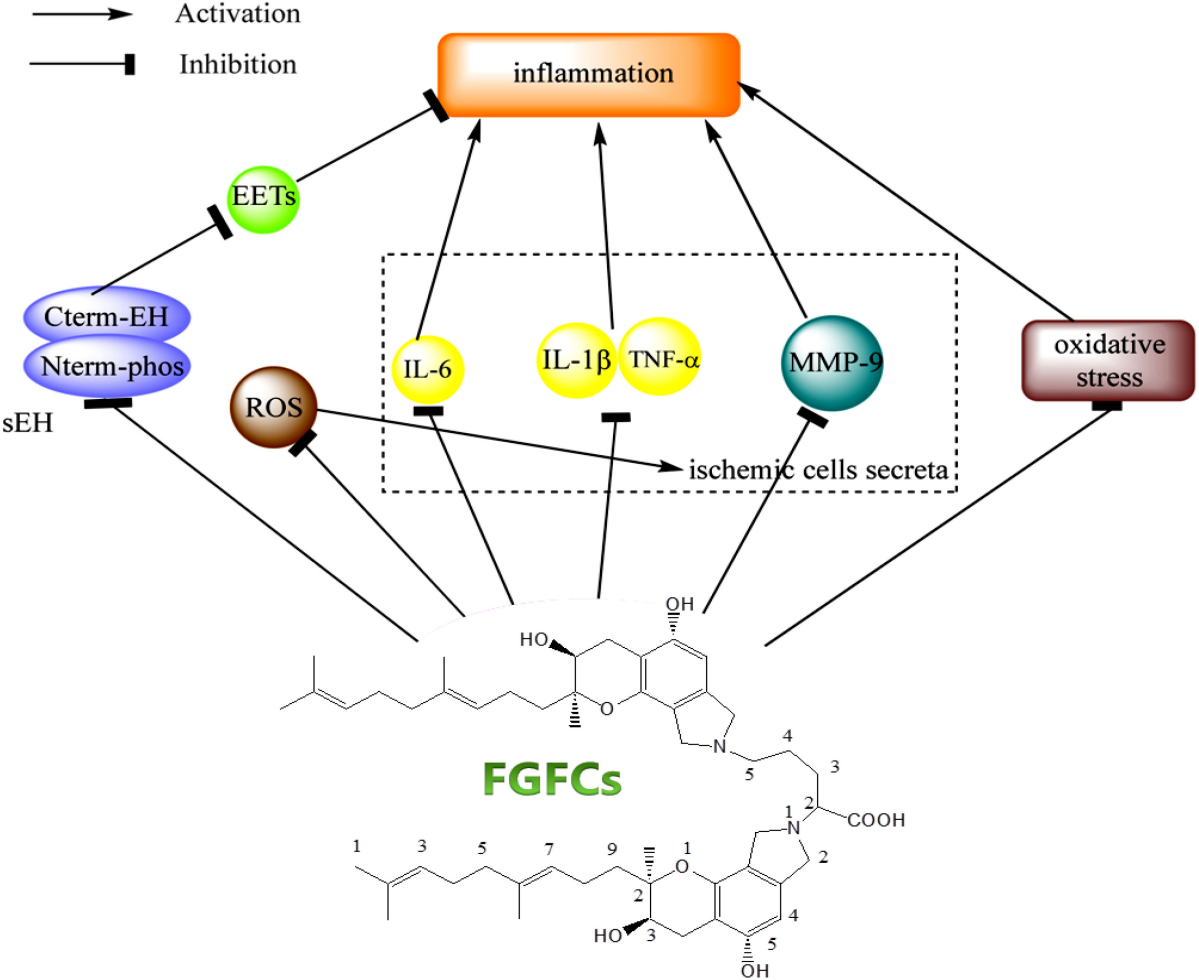
The signaling mechanism of FGFCs in Inflammatory disease (Hang et al., 2022).

**Figure 8:**
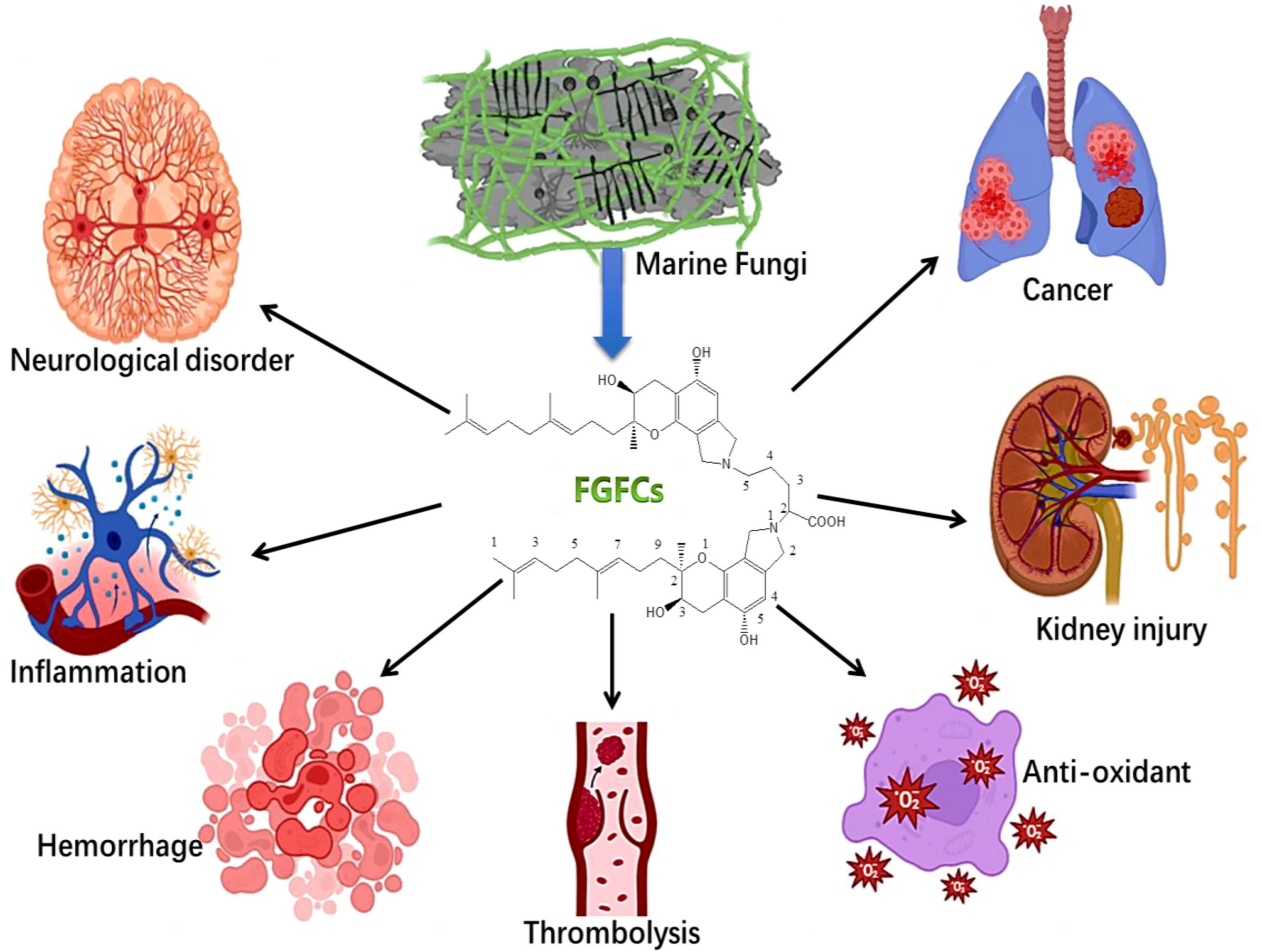
Overall approaches of FGFCs in Biomedical applications.

The secretion of (pro)inflammatory cytokines like IL-1β, IL-6 and TNF-α from ischemic cells was stimulated by reactive oxygen species production in occluded vessels reperfusion. As a consequence, these cytokines subsequently damage the cerebral vessels through hemorrhage and inflammation (Tsivgoulis et al., 2023). A previous study demonstrated that treating cerebral infarction mice model with FGFC1 (10 mg/kg) reduced the formation of hemorrhage and inflammation compared to t-PA treatment (10 mg/kg), which was due to decreasing the production of IL-1β, TNF-α, and IL-6 mRNA with the treatment of FGFC1 at 3 h after ischemia (Shibata et al., 2010). To support this finding, another study reported that mRNA expression of S100 calcium-binding protein A8, MMP-9, activator of transcription 3 and signal transducer, and IL-6 was downregulated by FGFC1 (Hashimoto et al., 2014). Ito et al. reported the inhibitory effect of FGFC1 in MMP-9 production using a mice model (Ito et al., 2014) and the oxidative stress inhibitory effect of FGFC1 was reported by Huang et al (Huang et al., 2018a). Akamatsu et al. observed hydroethidine signals responsible for ROS production in cerebral ischemia after 2 h reperfusion (Akamatsu et al., 2011) and the FGFC1 treatment decreased the hydroethidine signals, MMP-9 production and nitrotyrosine, thereby minimizing the inflammatory complication in cerebral infarction (**Figure 7**) (Rosell et al., 2008; Shibata et al., 2010). Overall, the FGFCs isolated from marine fungi could be the potential candidates for treating various diseases such as cancer, kidney injuries, neurological disorders, thrombolysis and hemorrhage (**Figure 8**).

## 6 Conclusion and perspectives

Overall, the review disclosed the fundamental background, different therapeutic concepts and potential application of FGFCs. Overall the review concluded that 1) FGFCs can be easily extracted from different types of marine fungi, 2) based on the types of derivatives, the FGFCs have different biological activities, 3) FGFC is the potential therapeutic drugs for treating several disorders related to inflammatory response, neural degenerative (cerebral infarction), kidney injury, hemorrhage, and thrombolysis. Based on the available scientific empirical evidence, FGFC is potentially used in treating diseases like edema, embolic stroke, ischemic stroke, renal ischemia, brain injury, cerebral infarction, acute kidney injury, cerebral artery occlusion, etc. Due to the specific targeting of receptors and signaling mechanism, FGFCs have proved to be extraordinary drugs in several therapeutic applications. However, the major limitations of FGFCs are lack of research on novel biomaterial platforms for efficient drug delivery in biomedical applications and also the efficiency of FGFCs should be investigated in deep and sound to use in other major complications such as diabetes, cardiovascular diseases, liver cirrhosis, skin and wound healing, and so on.
